# Hydroxyapatite-Tethered Peptide Hydrogel Promotes Osteogenesis

**DOI:** 10.3390/gels8120804

**Published:** 2022-12-08

**Authors:** Hongwen Yu, Jiaqi Song, Xianpeng Zhang, Kuo Jiang, Hong Fan, Yibing Li, Yuanting Zhao, Shichang Liu, Dingjun Hao, Guanying Li

**Affiliations:** 1 The Second Clinical Medical School, Shaanxi University of Chinese Medicine, Xianyang 712046, China; 2 Honghui Hospital, Xi’an Jiaotong University, Xi’an 710054, China; 3 School of Basic Medical Sciences, Xi’an Jiaotong University, Xi’an 710061, China

**Keywords:** hydrogel, peptide assembly, hydroxyapatite, osteoinductive, mimetic bone matrix, bone regenerative engineering

## Abstract

Hydroxyapatite (HAp) as natural bone composition is highly osteoinductive. To harvest its osteoinductivity in bone regenerative engineering, the HAp-supporting hydrogel is urgently needed to minimize inhomogeneous aggregation of HAp. Here, we developed a HAp-stabilizing hydrogel based on peptide self-assembly. FmocFFRR was efficient for HAp-capping due to arginine-phosphate interaction. Tethering FmocFFRR on the HAp surface facilitated self-assembly to form FmocFFRR/HAp hybrid hydrogel, enabling stable dispersion of HAp in it. The molecular interactions between FmocFFRR and HAp particles were studied using microscopic and spectral characterizations. FmocFFRR/HAp hydrogel exhibited more enhanced mechanical properties than FmocFFRR. The biocompatibility of FmocFFRR/HAp hydrogel was verified using an ATP assay and live-dead staining assay. More importantly, FmocFFRR/HAp hydrogel not only enabled cell attachment on its surface, but also supported 3D cell culturing inside the hydrogel. Further, 3D culturing of MC3T3-E1 preosteoblasts inside FmocFFRR/HAp hydrogel significantly enhanced the expressions of osteogenesis markers, including alkaline phosphate (ALP), type-I collagen (COL1), and osteocalcin (OCN), demonstrating the promoting effect of osteoblast differentiation. These findings inspire its potential application in bone regenerative engineering.

## 1. Introduction

Despite the successful clinical usage of autografts and allografts for bone substitution, repair, and augmentation [1], they are both plagued with their high cost, limited availability, and secondary damage at the donor site. Bone regenerative engineering that uses a top-down approach to regenerate bone tissue has attracted increasing attention in repairing bone defects [2]. One of the key aspects of bone regenerative engineering is the osteoinductive properties of the regenerative scaffolds or incorporating materials [3]. Hydroxyapatite (HAp) constitutes the major inorganic component in natural bones. Nanostructured HAp particles possess high osteoinductivity [4,5,6] and have been widely used in bone regenerative engineering [7,8]. However, HAp is very stable with low solubility in physiological conditions. Nanosized HAp tends to aggregate due to its high surface energies [9], impeding its osteoinduction efficacy. What is worse, inhomogeneous aggregation of HAp is risky to bone malformation during bone regeneration [10].

Satisfying HAp stability in a three-dimensional (3D) scaffold greatly benefits from the advanced development of material design over the last decade [11,12]. Polymeric biomaterials including natural polymers [13,14,15] and synthetic polymers [10,16] have been extensively employed as HAp-supporting scaffolds. Compared with these polymeric scaffolds, supramolecular peptide hydrogels based on small molecular peptide assembly [17] show prominent biocompatibility and biodegradability without causing observable inflammation. Because of the noncovalent interactions that drive peptide assembly [18,19], peptide hydrogels are more adaptive and feasible as 3D scaffolds for tissue engineering than polymer-based scaffolds. More importantly, scaffolds embracing bioactive peptides as a mimetic extracellular matrix (ECM) [20] enable cell adhesion and regulate cell proliferation, cell motion, polarity, mechanotransduction, and ECM signaling cascades, which crucially affect cell differentiation during bone development. In this regard, self-assembling peptide hydrogels become promising candidates as osteoinductive scaffolds for bone regenerative engineering. 

Fluorenyl-9-methoxycarbonyl diphenylalanine (FmocFF), as one of the smallest building blocks [21], could self-assemble into a self-supporting hydrogel. However, this basic self-assembled hydrogel lacks HAp-binding capacity and bioactivity allowing cell attachment. To address these issues, a co-assembly strategy using multicomponent was employed. Ghosh et al. [22] incorporated Fmoc-arginine (FmocR) with FmocFF to form HAp-decorated hydrogel. Wang et al. [23] introduced a cell adhesive ligand Fmoc-arginyl-glycyl-aspartate (FmocRGD) and developed FmocFF/FmocRGD hydrogel for three-dimensional (3D) culturing of mesenchymal stem cells. Recently, Vitale et al. [24] developed a three-component assembled hydrogel composed of FmocFF/FmocS/FmocRGD, where FmocS was Fmoc-serine. This hydrogel enabled HAp-binding and cell adhesion, and promoted osteoclast differentiation of RAW264.7 macrophages, showing potential in bone tissue engineering. One of the major drawbacks of multicomponent assembled hydrogel is that the HAp trapping manner, their mechanical properties, and their biological activities strongly depend on their formulation. Their osteoinductivity for osteoblast differentiation has not yet been explored.

**Figure 1:**
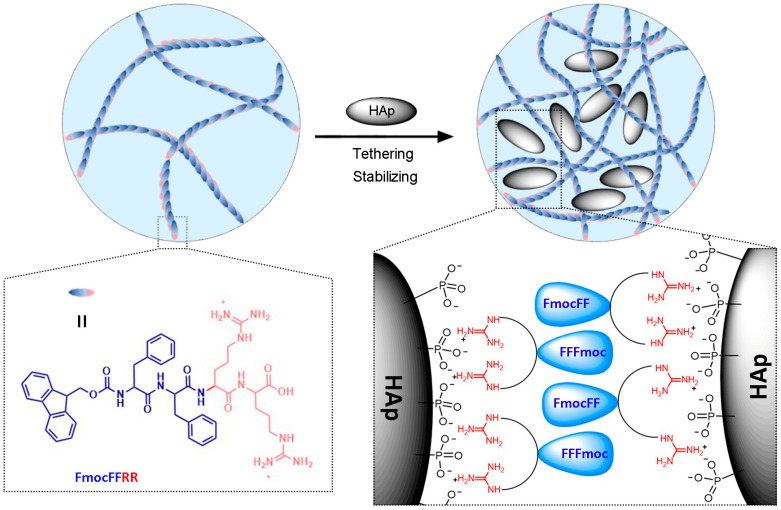
Schematic illustration of constructing FmocFFRR-tethering HAp hybrid hydrogel for HAp-stabilization.

To better harvest the osteoinductivity of HAp in bone regenerative engineering, in this work, we seek to construct Hap-stabilizing hydrogel composed of single peptide assembly for accurate guidance of Hap-induced osteogenesis. Recently, we reported a pentapeptide FmocFFRRR with high affinity to phosphate. The side chain of arginine residues has a pKa value of 12.48, indicating guanidine groups are protonated and possess positive charges under physiological condition. By adding phosphates, FmocFFRRR formed a self-assembling hydrogel, assisting the in situ mineralization of CaP [25]. When HAp that is rich in phosphate groups was added, however, FmocFFRRR was prone to form aggregation or microgels, probably due to the strong binding affinity. Herein, we compromised its HAp binding by reducing an arginine residue. The obtained tetrapeptide FmocFFRR possessed a balanced hydrophobic building block (FmocFF−) and hydrophilic residue (-RR), and self-assembled into nanofilaments in aqueous. We speculate that HAp is capable of tethering FmocFFRR via electrostatic phosphate–guanidine interaction [26]. Anchoring FmocFFRR on the HAp surface not only reduces the intermolecular distance of self-assembling building blocks that facilitates their self-assembly, but also promotes interfibrillar tethering, generating a HAp-trapping fibrous network for stable HAp dispersion (Figure 1). The mechanical properties, biocompatibility, and osteoinductive properties of HAp-tethered FmocFFRR assembling hydrogel were evaluated.

## 2. Results and Discussion

### 2.1. Synthesis and Characterizations

FmocFFRR was synthesized by standard solid phase peptide synthesis (SPPS) using 2-chlorotritylchloride resin and Fmoc-protected amino acids (Scheme S1). After cleavage from resin using trifluoroacetic acid (TFA), the crude product was precipitated in ether and purified on a semi-prepared HPLC to obtain the target peptide with a 97.7% purity (Figure S1). Structural characterizations were carried out using a high-resolution mass spectrum (Figure S2) and ^1^H NMR spectrum (Figure S3), confirming the production of target peptide FmocFFRR.

### 2.2. Formation of HAp-Anchoring Hydrogels

**Figure 2:**
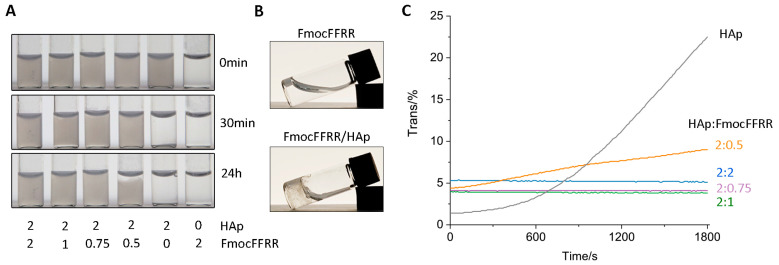
(**A**) Optical images of 2 mg/mL HAp particles dispersed in FmocFFRR solution in ddH_2_O at different concentrations (0, 0.5, 0.75, 1.0, 2.0 mg/mL) for 0 min, 30 min, or 24 h. (**B**) Optical images of 2 mg/mL FmocFFRR in the absence or presence of HAp particles (4 mg/mL) in ddH_2_O. (**C**) Transmittance curves of 2 mg/mL HAp dispersed in ddH_2_O or in FmocFFRR assemblies at 0.5, 0.75, 1.0, or 2.0 mg/mL over a 30 min period. Transmittance was measured at 600 nm.

FmocFFRR dissolved well in water and formed viscous fluid at low concentrations (0.5, 1.0, and 2.0 mg/mL). When increasing its concentration (4.0 and 8.0 mg/mL), it formed a transparent, self-supporting hydrogel (Figure S4), suggesting the self-assembly of FmocFFRR. To investigate whether FmocFFRR self-assembly could immobilize HAp, we dispersed nanosized HAp particles into FmocFFRR fluids at different concentrations. As shown in Figure 2A, HAp rapidly precipitated within 30 min in the absence of FmocFFRR assembly. In very low concentrations of FmocFFRR (0.5 mg/mL), HAp particles could disperse well for hours, and partial precipitation was observed after 24 h. In FmocFFRR assembly higher than 0.5 mg/mL, HAp stood well dispersive and formed opaque mixtures that were stable even after 48 h (Figure S5), suggesting high stability of HAp on the FmocFFRR/HAp mixture. In addition, FmocFFRR at 2.0 mg/mL successfully gelated into self-supporting hydrogel after being tethered by HAp particles, compared to fluidic FmocFFRR in the absence of HAp (Figure 2B). It suggested that the addition of HAp particles significantly facilitated FmocFFRR assembly, because anchoring of FmocFFRR on the HAp surface could (1) neutralize its positive charges on the arginine residues overcoming charges repulsion, (2) reduce intermolecular distance of self-assembly building blocks, and (3) tether self-assembled filaments into the dense fibrous network. The stability of HAp dispersion was further quantified by measuring transmittance at 600 nm (Figure 2C). The results showed that the HAp/FmocFFRR mixture with a weight ratio higher than 2:0.75 kept opaque with low transmittance over 30 min period. Therefore, FmocFFRR assembly could efficiently stabilize HAp dispersion in the hydrogel.

**Figure 3:**
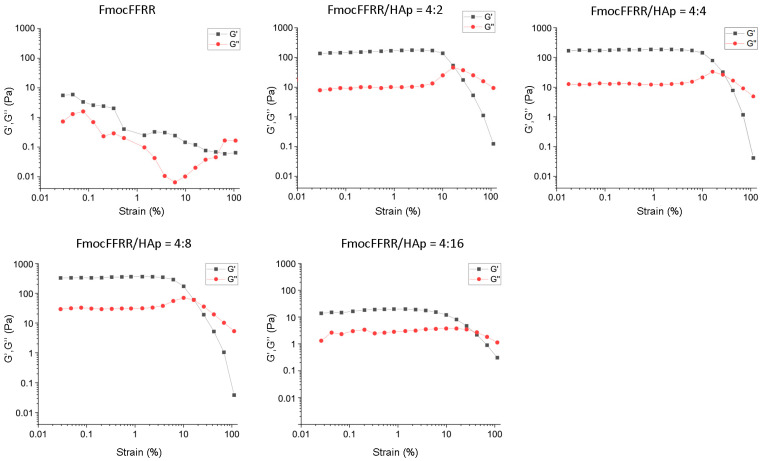
Strain amplitude sweep tests of 4 mg/mL FmocFFRR containing 0, 2, 4, 8, or 16 mg/mL HAp particles at a fixed angular frequency (1.0 rad/s) at room temperature.

We analyzed the mechanical properties of HAp/FmocFFRR-tethering hydrogel using rheology. Oscillatory strain-sweep rheological analysis (Figure 3) showed that 4 mg/mL FmocFFRR assembly in the absence of HAp possessed low storage modulus (G’) and loss modulus (G″) values with a breaking strain at 47%, indicating the formation of a weak hydrogel. When mixing with 2 mg/mL HAp, FmocFFRR formed a strong gel that showed remarkably increased G’ and G″ values with a wide linear viscoelastic region (LVR). In a 4:4 (*w*/*w*) FmocFFRR/HAp hydrogel, both G’ (180 Pa) and G″ (closed to 10 Pa) values were similar, while in a 4:8 (*w*/*w*) FmocFFRR/HAp hydrogel, the G’ value was a two-fold increase and G’’ value was a three-fold increase, compared to the 4:2 (*w*/*w*) FmocFFRR/HAp hydrogel. The incorporation of HAp into FmocFFRR assembly significantly enhanced its mechanical properties, in line with Ghosh’s [22] and Vitale’s findings [24]. It is reasonable to postulate that the interaction between FmocFFRR and HAp contributes to fibers crosslinking and strengthens the fibrous entanglements. When the composition of HAp increased to 16 mg/mL, however, the FmocFFRR/HAp hydrogel became weakened with reduced G’ and G″ values. It is not surprising, because oversaturation of HAp reinforces peptide binding to their surfaces, thus weakening fiber-fiber interaction. These results demonstrated a 1:2 (*w*/*w*) FmocFFRR/HAp formulate to fabricate a relatively strong hydrogel.

### 2.3. Structural Characterizations of FmocFFRR/HAp Hydrogels

**Figure 4:**
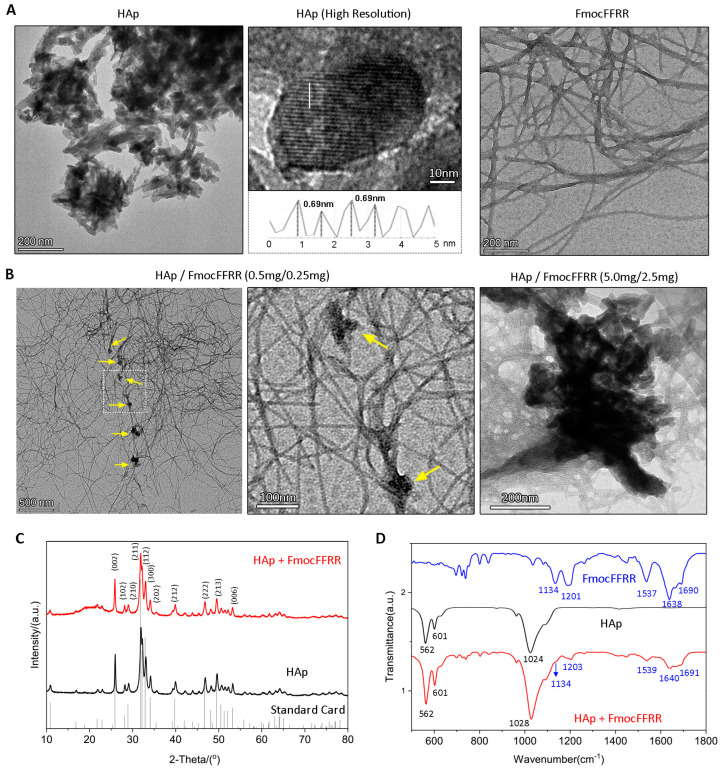
(**A**) TEM images of HAp particles (5.0 mg/mL) with high-resolution image (middle panel) showing lattice spaces. The gray value distribution curve of indicated white light was also showed to indicate the interlamellar distance. TEM image of FmocFFRR assembly (2.5 mg/mL) was shown at the right panel. (**B**) TEM images of HAp/FmocFFRR hydrogel at the concentration of 0.50/0.25 (mg/mL) or 5.0/2.5 (mg/mL) in Tris buffer. Yellow arrows indicated peptide-bound HAp particles. (**C**) XRD patterns of free HAp particles (black) and the lyophilized HAp/FmocFFRR sample (red). A standard card of the calculated XRD pattern of HAp was also shown for comparison. (**D**) FTIR spectra of FmocFFRR (blue), HAp (black), and HAp/FmocFFRR (red).

After formulating the compositions of the tough HAp/FmocFFRR hydrogel, we examined their microstructures using transmission electron microscopy (TEM). As shown in Figure 4A, HAp particles used in these tests showed nanorod-like structures. High-resolution TEM image revealed the crystalline structure of HAp nanoparticles with a lamellar distance of 0.69 nm, which was attributed to the crystal lattice *c* parameter [27,28]. FmocFFRR self-assembled into nanofibers with a diameter of 4 nm. When FmocFFRR was mixed with HAp, it formed a dense fibrous network. At a low concentration of mixed gel (HAp/FmocFFRR = 0.50/0.25, mg/mL), we could clearly observe individual HAp particles surrounded by the peptide assembling nanofibers (Figure 4B), indicating peptide bound to HAp. HAp clustering was observed at a high concentration of mixed gel (HAp/FmocFFRR = 5.0/2.5, mg/mL). However, the edge of HAp clusters was fused with fibrous bundles. These observations suggested HAp tethered FmocFFRR assembling nanofibers, which in turn stabilized HAp dispersion. X-ray diffraction (XRD) analysis (Figure 4C) demonstrated that the FmocFFRR/HAp mixture showed a similar XRD pattern compared with free HAp particles. This result suggested that the tether of FmocFFRR on the HAp surface did not affect its crystalline structure.

FT-IR spectra were further conducted to demonstrate the molecular interaction between HAp and FmocFFRR. The results (Figure 4D) showed that the phosphate stretching vibration at 1024 cm^−1^ of HAp slightly shifted to 1028 cm^−1^ after anchoring of FmocFFRR. Slight shifts of guanidine groups vibration (1537 cm^−1^) and amide bands (1638 cm^−1^ and 1690 cm^−1^) of FmocFFRR after HAp/FmocFFRR interaction were also observed. The intensity of the amide I band at 1690 cm^−1^ assigned as β-turn [29] of FmocFFRR assembly increased after HAp/FmocFFRR interaction compared to the amide II band internally at 1638 cm^−1^, in accordant with our previous finding on FmocFFRRR/CaP minerals interaction [25]. These results suggested HAp/FmocFFRR interaction promoted the molecular assembly of FmocFFRR.

### 2.4. Biocompatibility of FmocFFRR/HAp Hydrogels

**Figure 5:**
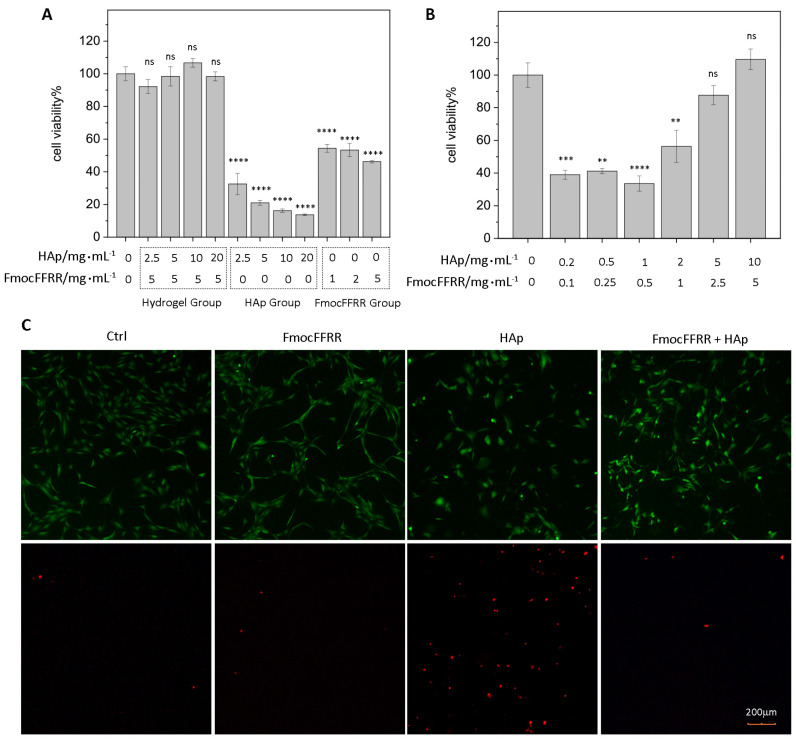
(**A**) MC3T3-E1 cell suspensions were mixed with freshly prepared FmocFFRR/HAp mixture for in situ gelations (hydrogel group), with free HAp particles (HAp group), or with FmocFFRR assemblies (FmocFFRR group). For the controlled groups, MC3T3-E1 cell suspensions were mixed with culture media. Cells were allowed for cultured for another 48 h and the cell viability was evaluated. (**B**) Cell viability of MC3T3-E1 cells after culturing in FmocFFRR/HAp hydrogels at different concentrations with a fixed 1:2 weight ratio. ns: non-significant; **: *p* < 0.01; ***: *p* < 0.001; ****: *p* < 0.0001. (**C**) Live-dead staining of MC3T3-E1 cells after culturing in FmocFFRR/HAp (5.0/10.0 mg/mL) hydrogel, or treated with free HAp particles (10.0 mg/mL), FmocFFRR assemblies (5.0 mg/mL) for 48 h. The controlled cells were seeded on a tissue-culture plate. Green channels indicated live cells; red channels indicated dead cells, respectively.

To evaluate the osteoinductive potency of FmocFFRR/HAp hydrogels, we first examined their biocompatibility. Preosteoblast MC3T3-E1 cells, a typical cell model for studying osteogenesis [30], were seeded on the culture plate that was previously coated with FmocFFRR/HAp hydrogel. The live-dead cell staining results (Figure S6) showed that a large number of MC3T3-E1 cells attached on the hydrogel surface without inducing much cell death, suggesting great cell adhesion of FmocFFRR/HAp hydrogel. MC3T3-E1 cells grew on a 96-well plate that was pretreated with free HAp particles, free FmocFFRR assemblies, and HAp/FmocFFRR hydrogels for 48 h, and the viability was further quantified using an ATP assay. As shown in Figure 5A, free HAp nanoparticles exhibited high cytotoxicity against MC3T3-E1 cells. To reason their cytotoxicity, we observed the microscopic images of HAp-treated MC3T3-E1 cells and found that HAp aggregates strongly attached to cell surfaces (Figure S7), which may affect cell functions and contribute to the cytotoxicity. FmocFFRR assemblies without tethering on HAp showed moderate cytotoxicity, with less than 50% cell viability at a concentration of 5mg/mL. Excitingly, when HAp and FmocFFRR mixed to form hydrogels, they showed significant biocompatibility with cell viability greater than 90%. The 1:2 (*w*/*w*) FmocFFRR/HAp formulating hydrogel at 5/10 mg/mL concentration exhibited higher cell viability than the non-treated cells, suggesting a promoting effect on cell proliferation. This was reasoned that the 1:2 (*w*/*w*) ratio of FmocFFRR to HAp formed the stable hydrogel (Figure 3) with a minimum amount of free HAp or free FmocFFRR compositions leaked into culture media. The cytotoxicity of 1:2 (*w*/*w*) FmocFFRR/HAp hydrogel at different amount concentrations was also conducted. The results (Figure 5B) demonstrated that at low content of FmocFFRR (0.1, 0.25, 0.5, and 1.0 mg/mL), the FmocFFRR/HAp mixture exhibited a cytotoxic effect with cell viability less than 40%. With a high content of FmocFFRR (2.5, 5.0 mg/mL) that enabled stable FmocFFRR/HAp hydrogel formation, the hydrogel was of great biocompatibility with cell viability greater than 80%. Non-covalent interactions, including *π*-*π* stacking, H-bonds, and electrostatic interactions, drove the formation of the FmocFFRR/HAp hybrid hydrogel. To evaluate whether FmocFFRR/HAp hydrogel would dissemble to leak FmocFFRR and HAp particles that show moderate cytotoxic effect in this study, we incubated the hydrogel with MC3T3-E1 cells which were attached to the culture plate. The results (Figure S8) showed that the leaching contents showed comparable vital cell numbers and a few numbers of dead cells compared to the controlled experiment.

To further investigate the capability of cell encapsulation, a mixture of FmocFFRR/HAp was in situ gelated with MC3T3-E1 cell suspension in a basal culture medium and kept in a cell-cultured condition overnight. Hoechst 33,258 staining indicated the existence of MC3T3-E1 cells inside the hydrogel (Figure S9). Live-dead staining of encapsulated cells was performed. The results (Figure 5C) suggested that most of the encapsulated cells inside the FmocFFRR/HAp hydrogel were alive (stained green) with a few cells stained red (indicating dead cells), comparable with controlled cells attached to the cell-cultured plate. Notably that portion of live cells encapsulated in the hydrogel remained round in shape, assigning to their 3D culturing environment. In contrast, cell suspension that was treated with free FmocFFRR was capable of attaching to the culture plate and showed little dead cells. However, the cell number was less than the non-treated group, suggesting FmocFFRR affected the proliferation of MC3T3-E1 cells. Cell suspension treated with free nanosized HAp particles showed large numbers of dead cells, suggesting the cytotoxic effect against the MC3T3-E1 cell. These data agreed with the results of the ATP assay (Figure 5A).

### 2.5. Osteoinductive Properties of FmocFFRR/HAp Hydrogels 

**Figure 6:**
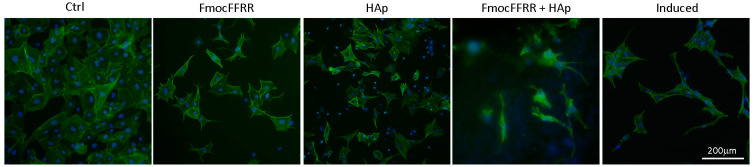
Morphological analysis of MC3T3-E1 cells after culturing in FmocFFRR/HAp (5.0/10.0 mg/mL) hydrogel, free HAp particles (10.0 mg/mL), FmocFFRR assemblies (5.0 mg/mL), or treated with osteoinduction medium (Induced) for 5days. F-actin filaments were stained with Alexa Fluor 488-conjugated phalloidins, and nuclear were stained with DAPI (blue).

MC3T3-E1 preosteoblasts exhibit a high level of osteoblast differentiation into mature osteocytes after growing in an osteoinduction medium that usually contains *L*-ascorbic acid and phosphate sources. When the MC3T3-E1 preosteoblasts were maintained in the FmocFFRR/HAp hydrogel for culturing in the basal DMEM media without supplementary *L*-ascorbic and phosphate, we observed that cell bodies elongated and started growing dendrites after 5 days, similar to those treated with osteoinduction medium (Figure 6). This finding implied that the FmocFFRR/HAp hydrogel potentially induced osteogenesis of preosteoblasts. MC3T3-E1 cells treated with free FmocFFRR exhibited irregular morphologies, meanwhile treatment of free HAp particles induced preosteoblasts body shrinkage and nuclear condensation, suggesting induced cytotoxicity accordantly with previous results. The controlled MC3T3-E1 cells remained in fibroblast shape without the treatment of osteoinduction medium.

**Figure 7:**
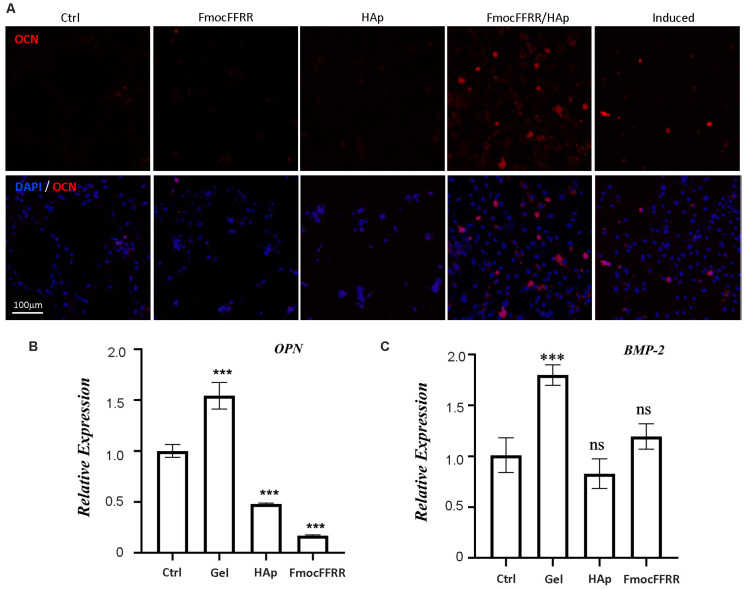
MC3T3-E1 cells were cultured with basal culture medium in FmocFFRR/HAp (5.0/10.0, mg/mL) hydrogel, free HAp particles (10.0 mg/mL), FmocFFRR assemblies (5.0 mg/mL), or treated with osteoinduction medium (Induced) for 7 days. (**A**) Protein expression of OCN was immunostained with an anti-OCN antibody. Cell nuclear was also stained with DAPI. Gene expression of osteogenesis markers OPN (**B**) and BMP-2 (**C**) was quantified using qRT-PCR. The expression of GAPDH was used as an internal standard. Data were shown as mean ± SD, *n* = 6; *** *p* < 0.001.

Osteoblast differentiation of 3D culturing preosteoblasts in FmocFFRR/HAp hydrogel was further confirmed by assessing the expression of osteogenesis biomarkers. After 7 days culture of MC3T3-E1 cells in the FmocFFRR/HAp hydrogel without supplementary *L*-ascorbic and phosphate, the expression of alkaline phosphatase (ALP) and type-I collagen (COL1) as biomarkers for early-stage osteoblast differentiation [31] was evaluated using immunostaining fluorescent staining. Compared to control MC3T3-E1 cells that were cultured with basal culture medium, HAp-treated cells showed elevated expression of ALP (Figure S10), and FmocFFRR assembly-treated cells showed slightly increased expression of COL1 (Figure S11). However, HAp treatment or FmocFFRR assembly treatment of MC3T3-E1 cells induced a negligible change of osteocalcin (OCN) expression (Figure 7A), a commonly used biomarker for late-stage osteoblast differentiation [31]. These results suggested that sole treatment with FmocFFRR assemblies or HAp particles could induce the osteoblasts differentiation into the early stage for ECM secretion and maturation. In comparison, 3D culturing of MC3T3-E1 cells in FmocFFRR/HAp hydrogel significantly promoted the expression of osteogenesis markers including ALP, COL1, and OCN, suggesting a late stage of osteoblasts differentiation occurred for bone matrix mineralization. MC3T3-E1 cells that were induced with osteoinduction medium for 7 days exhibited increasing expressions of ALP, COL1, and OCN. When MC3T3-E1 cells were 3D cultured in FmocFFRR/HAp hydrogel, higher expression levels of COL-1 and OCN were observed compared to the induced cells by osteoinduction medium, revealing FmocFFRR/HAp hydrogel has a higher osteoinductivity than osteoinduction medium used in this study.

The RNA level of osteopontin (OPN) expression, another biomarker for late-stage osteoblast differentiation, was quantified by qRT-PCR. The results (Figure 7B) demonstrated that 3D culturing in hydrogel promoted the OPN level, while the treatment of HAp or FmocFFRR assembly reduced its expression. MC3T3-E1 cells cultured in hydrogel also exhibited increasing RNA levels of bone morphogenic protein 2 (BMP-2) (Figure 7C), which positively regulates osteogenesis processes [32]. Taking into consideration the morphological changes of preosteoblasts and the increasing level of both protein expressions and RNA expressions of osteoblast differentiation-related markers, we could conclude that FmocFFRR/HAp hydrogel capably supported 3D culturing of preosteoblasts, and promoted their osteoblast differentiation, exhibiting great potentials as a highly osteoinductive scaffold in bone regenerative engineering.

Despite the regulatory effects of both positively charged arginine and negatively charged aspartic acid or glutamic acid on HAp mineralization [33,34], recent material-design for HAp-supporting scaffolds focused on polycarboxylate-rich scaffold materials as HAp capping [10,35]. We demonstrated an arginine-containing peptide as an alternative to HAp-stabilizing materials. The HAp-anchorable peptide FmocFFRR was designed by tailoring an arginine residue to a self-assembling building block FmocFF. Anchoring FmocFFRR on the HAp surface through electrostatic guanidine–phosphate interaction facilitated the self-assembly of FmocFFRR, due to the neutralization of charge repulsion, the reduction of intermolecular distance between building blocks, and the tether of self-assembling filaments into the dense fibrous network. On the other hand, FmocFFRR assembling on the nanosized HAp surface reduced its surface energy and stabilized its dispersion in the scaffold.

Nanosized HAp particles are reported biocompatible [10,22,24]. In this study, however, we used commercially available HAp particles with an average size of 20 nm and found that they exhibited cytotoxic effect against MC3T3-E1 preosteoblasts. Strong attachment of them to the cell surfaces (Figure S7) is one of the factors contributing to their cytotoxicity. An assembling peptide FmocFFRR was not cytotoxic but showed moderate anti-proliferative effect (Figure 5C). Mixing FmocFFRR and HAp at a ratio of 1:2 (*w*/*w*) in a high concentration (FmocFFRR > 2.5 mg/mL) generated a stable hybrid hydrogel, which surprisingly turned over the cytotoxicity of single components and exhibited high biocompatibility. This action is similar in many cases of polymeric hydrogels. For example, methacryloyl monomer and initiators are known to be harmful. They polymerize in situ to form polymeric hydrogels with less cytotoxicity. Non-covalent interactions including *π*-*π* stacking, H-bonds, and electrostatic interactions are the major forces that drive the formation of FmocFFRR/HAp hydrogel. However, the electrostatic interaction between arginine and phosphate is very strong and considered “covalent-like” [26,36]. This feature endows FmocFFRR/HAp hydrogel with polymer-like properties showing high stability and biocompatibility.

Despite the cytotoxic effect of nanosized HAp particles we used in this study, they induced an increasing expression level of ALP (Figure S10). FmocFFRR itself promoted the secretion of COL1 (Figure S11), which participates in the bone ECM construction. MC3T3-E1 preosteoblasts cultured in the FmocFFRR/HAp hydrogel not only showed lifted levels of ALP and COL1, but also higher expression of OCN than that induced by osteoinduction medium, suggesting that FmocFFRR/HAp hydrogel is highly osteoinductive. The Uesugi group [37,38] reported positively charged self-assembling molecules could selectively bind to heparan sulfate and promote clustering of heparan sulfate proteoglycans (HSPGs) such as syndecan-4. It is reasonable to hypothesize that FmocFFRR binds to heparan sulfate. Therefore, FmocFFRR/HAp hydrogel may involve in heparan sulfate-growth factors related signaling for promoting osteoblast differentiation.

## 3. Conclusions

In summary, we demonstrated a HAp-supporting hydrogel based on peptide assembly. FmocFFRR bound to HAp through strong electrostatic interactions between arginine residues of FmocFFRR and phosphate groups on the surface of HAp. HAp-tethering FmocFFRR facilitated self-assembly and crosslinking to form FmocFFRR/HAp hybrid hydrogel, mimicking the natural bone ECM structure. The 1:2 (*w*/*w*) ratio of FmocFFRR/HAp mixture at a high concentration (FmocFFRR > 2.5 mg/mL) formed stable hydrogel with enhanced mechanical properties and high biocompatibility. Both the ATP assay and live-dead staining assay verified the formulating FmocFFRR/HAp hydrogel showed negligible cytotoxicity. More importantly, FmocFFRR/HAp hydrogel not only enabled cell attachment on its surface, but also supported 3D cell culturing inside the hydrogel to promote the osteoblast differentiation of MC3T3-E1 preosteoblasts, inspiring its potential application in bone regenerative engineering. These findings not only expand the diversity of osteoinductive hydrogels for osteogenesis, but also provides a bottom-up approach for fabricating ECM-mimicking hydrogel with high accuracy. We believe that this strategy can be programmed with multifunctionalities and broadens its appeal.

## 4. Materials and Methods

### 4.1. Materials and Instruments

All the solvents and chemicals were commercially available and used as received without further purification. HAp nanoparticles with an average size of 20 nm were purchased from Macklin Co., Ltd. (Shanghai, China). Mouse Embryonic Osteoblasts Cell (MC3T3-E1) was purchased from the American Type Culture Collection (ATCC). Dulbecco’s modified Eagle medium (DMEM), fetal bovine serum (FBS), and penicillin-streptomycin liquid (100X) were purchased from Gibco (Waltham, MA, USA). The Calcein-AM/PI live and dead cell double stain kit was purchased from Solarbio Technology Co., Ltd. (Beijing, China). The primary antibody of OCN was purchased from Boster Biological Technology Co., Ltd. (Pleasanton, CA, USA); primary antibodies of ALP, and Col1 were purchased from Proteintech Group Inc, Ltd. (Chicago, IL, USA). Cy5-conjugated secondary antibody was purchased from Boster Biological Technology Co., Ltd. (Pleasanton, CA, USA). CellTiter-Glo^®^ Luminescent Cell Viability Assay was purchased from Promega Co., Ltd. (Madison, WI, USA). PCR primers were purchased from Sangon Biotech Co, Ltd. (Shanghai, China). SPARK easy Cell RNA Kit, SPARK script ‖ RT Plus kit, SYBR Green qPCR Master Mix were purchased from SparkJade Biological Technology Co., Ltd. (Shandong, China). NucBlue Live ReadyProbe, NucBlue Dead ReadyProbe, and ActinGreen 488 ReadyProbe reagents were purchased from Thermo, Ltd. (Waltham, MA, USA).

The mass spectrum of FmocFFRR was measured by Agilent-6125B ESI mass spectrometer (Agilent, Santa Clara, CA, USA), and the purity was checked on an EClassical^®^ 3140 HPLC (Elite, China). ^1^H spectrum was acquired on a 400 MHz JEOL spectrometer (JEOL, Tokyo, Japan). The ^1^H NMR chemical shifts (δ) were given in ppm referring to internal standard tetramethylsilane (TMS). The transmittance of the FmocFFRR/HAp mixture was recorded on an Ultraviolet-visible Spectrophotometer (YOKE L6). The rheology data were collected using a HAAKE MARS rheometer (Thermo) with an environmental test chamber. TEM images were obtained using a Talos L120C transmission electron microscope. X-ray diffractometry utilized Bruker AXS D8 diffractometer (Bruker, Billerica, MA, USA) using a copper Kα (λ = 1.5406 Å) radiation source. FTIR spectra were recorded using a Bruker Vertex 70 FTIR spectrophotometer (Bruker, USA) in an attenuated total reflectance (ATR) mode and analyzed through OriginPro 2021 Software Student Version (Originlab, Northampton, MA, USA). Fluorescence images were captured on a DMi8 fluorescent inverted microscope (Leica, Wetzlar, Germany). qRT-PCR was performed on a QuantGene 9600 real-time PCR (Bioer, Hangzhou, China). 

### 4.2. Synthesis

Peptide FmocFFRR was designed and synthesized by solid phase peptide synthesis (SPPS) using 2-chlorotritylchloride resin and Fmoc-protected amino acids (Scheme S1). The crude product was purified on an EClassical P3140 HPLC equipped with a SinoChrom C18 column (ODS-BP, 10 μm, 20.0 mm × 250 mm) to obtain white powders. Yield 52.1%. The purity was checked as 97.7%, using an EClassical P3140 HPLC equipped with a SinoChrom C18 column (ODS-BP, 5 μm, 4.6 mm × 250 mm). ESI-MS (*m*/*z*) calcd. for C45H54N10O7, 846.9900, found [M + 2H]^2+^ 424.2444 and [M + H]^+^ 847.4774. ^1^H NMR (400 MHz, DMSO-d6) (ppm): δ 8.22 (dd, *J* = 14.8, 7.7 Hz, 2H), 8.09 (d, *J* = 8.0 Hz, 1H), 7.88 (d, *J* = 7.7 Hz, 2H), 7.65–7.51 (m, 4H), 7.41 (ddt, *J* = 7.4, 4.2, 2.2 Hz, 2H), 7.33–7.14 (m, 12H), 4.70–4.54 (m, 1H), 4.37–4.31 (m, 1H), 4.27–4.15 (m, 3H), 4.15–4.04 (m, 2H), 3.19–3.01 (m, 5H), 2.95–2.79 (m, 2H), 2.73–2.65 (m, 1H), 1.82–1.66 (m, 2H), 1.66–1.45 (m, 6H).

### 4.3. Preparation of FmocFFRR/HAp Hydrogels

To a freshly prepared FmocFFRR solution in ddH_2_O was added HAp suspension in ddH_2_O and mixed with a pipette. The mixture was allowed to stand still for aging at room temperature.

### 4.4. Transmittance Measurements

First, 1 mL of freshly prepared FmocFFRR solution in ddH_2_O was placed in quartz, to which was added 1mL HAp suspension containing 4 mg HAp in ddH_2_O and mixed with a pipette. The quartz was immediately placed in the sample chamber of the UV-vis absorbance spectrometer. The transmittance was recorded at 600 nm for a 30 min period. The final weight ratio of FmocFFRR/HAp was (mg/mg) 0/2, 0.5/2, 0.75/2, 1/2, and 2/2, respectively.

### 4.5. Rheological Analysis

The mixtures of FmocFFRR/HAp containing 4 mg/mL of FmocFFRR and different amounts of HAp (0, 2, 4, 8, 16 mg/mL) were prepared and aged for 1 h at room temperature. Samples were cast between a 25 mm diameter stainless steel upper plate and a lower Peltier plate with the gap between plates at 1.0 mm for all measurements. Oscillatory strain sweeps (0.01–100% strain) at a fixed frequency of 1 Hz were performed.

### 4.6. Transmission Electron Microscopy (TEM)

A drop of 5 μL sample was added to the discharged copper grids coated with thin carbon film for about 60 s. After removing the excess solution with filter paper, the grids were washed with pure water and stained with 1% uranyl acetate for 30 s. The grid was allowed to dry at room temperature and ready for TEM imaging.

### 4.7. XRD Analysis

XRD diffraction patterns of HAp particles and lyophilized FmocFFRR/HAp sample were measured on a BrukerAXS D8 diffractometer using a copper Kα (λ = 1.5406 Å) radiation source. Scans were performed over a 2θ range of 10–80°.

### 4.8. FTIR Analysis

FTIR spectra of HAp particles, lyophilized FmocFFRR, or FmocFFRR/HAp mixtures were recorded using a Bruker Vertex 70 FTIR spectrophotometer in an attenuated total reflectance (ATR) mode and analyzed through OriginPro 2021 Software Student Version (Originlab, Northampton, MA, USA). The wavenumber range is 4000–400 cm^−1^.

### 4.9. Cell Culture

Pre-osteoblasts MC3T3-E1 cells were purchased from the American Type Culture Collection (ATCC, CRL-2593^TM^) and cultured in DMEM medium supplemented with 10% (*v*/*v*) FBS and 1% (*v*/*v*) penicillin–streptomycin. Cells were maintained in a fully humidified incubator containing 5% CO_2_ at 37 °C.

### 4.10. Cell Viability Analysis

DdH_2_O used in cellular experiments was filtered through a 0.22 μm pore-size membrane. Then, 40 μL of freshly prepared HAp suspension (2.5, 5.0, 10, or 20 mg/mL), FmocFFRR assembly (1.0, 2.0, or 5.0 mg/mL), FmocFFRR/HAp mixture containing various HAp contents (5.0/2.5, 5.0/5.0, 5.0/10, or 5.0/20 mg/mL), or 1:2 (*w*/*w*) FmocFFRR/HAp mixture at different concentrations (0.1/0.2, 0.25/0.5, 0.5/1.0, 1.0/2.0, 2.5/5.0, or 5.0/10 mg/mL) in ddH_2_O was added to a 96-well plate and was sterilized under UV light irradiation for 4 h at room temperature. Then, 100 μL of 1 × 10^5^ cells/mL MC3T3-E1 cell suspension was seeded to each well and cultured for 48 h. The ddH_2_O pre-coated wells were used as the control. The viability was assessed using a luminescence-based ATP assay following the manufacturer’s instruction. Briefly, 100 μL assay reagent was added to each well and mix the contents vigorously for 5 min to induce cell lysis. After maintaining the plate in the incubator for 15 min, 100 μL mixture in each well was transferred into a black plate. The luminescence was recorded immediately using a SuPerMax 3100 plate reader (Flash, Shanghai, China).

### 4.11. Cell Staining

MC3T3-E1 cell suspension was mixed with HAp, FmocFFRR of FmocFFRR/HAp mixture in DMEM media and placed seeded on a 24-well plate. Cell viability was assessed by Calcein-AM/PI staining following the manufacturer’s instruction. For staining the live cell nuclear of encapsulated MC3T3-E1 cells inside the FmocFFRR/HAp hydrogel, two drops of NucBlue Live ReadyProbe were added and stained at 37 °C for 30 min. After being washed with phosphate-buffered saline (PBS) three times, the samples were captured on a fluorescent inverted microscope. For staining F-actin of MC3T3-E1 cells, cells were fixed with 4% paraformaldehyde (PFA) in PBS for 15 min and permeabilized with 0.1% Triton X-100 in PBS for 5 min. Additionally, cells were treated with a drop of NucBlue Dead ReadyProbe and ActinGreen 488 ReadyProbe and stained at room temperature for 1 h. After being washed with phosphate-buffered saline (PBS) three times, the samples were captured on a fluorescent inverted microscope.

### 4.12. Immunofluorescent Staining

MC3T3-E1 cells were fixed with 4% PFA and permeabilized with 0.1% (*v*/*v*) Triton X-100 in PBS. After blocking with 5% (*w*/*v*) BSA for 1 h, the cells were incubated with an anti-osteocalcin antibody (1:100; PB1009), anti-collagen type-I antibody (1:100; 14695-1-AP), and anti-alkaline phosphatase antibody (1:100; ET1601-21) overnight at 4 °C. Subsequently, cells were washed three times with PBS and incubated with Cy3-coupled secondary antibody (1:1000; Abcam, UK) for 1 h, followed by incubation with DAPI for 15 min. Fluorescent signals were captured using a fluorescence microscope.

### 4.13. RNA Quantification

Total RNA was extracted using an RNA Purification Kit (SparkJade, China) and cDNA was obtained from 500 ng of total RNA using the Reverse Transcription Kit (SparkJade). qRT-PCR was performed using SYBR Green qPCR Master Mix (SparkJade). Relative gene expression was calculated using the 2^−ΔΔCT^ method, and GAPDH was used as a reference for normalization. The primers were purchased from BioTNT, and primer sequences for BMP-2, OPN, and GADPH were used as follows:

hBMP-2: 5′-CAGAACACAAGTCAGTGGGAGAGC-3′ (F), 5′-GAGGTGCCACGATCCAGTCATTC-3′ (R);
hOPN: 5′-ATGGACGACGATGATGACGATGATG-3′ (F), 5′-ATGGCTGCCCTTTCCGTTGTTG-3′ (R);
hGADPH: 5′-TGAACGGGAAGCTCACTGG-3′ (F), 5′-TCCACCACCCTGTTGCTGTA-3′ (R).

### 4.14. Statistical Analysis

All results were presented as mean and standard deviation with 3–6 independent experiments. The statistics were analyzed using the SPSS software. The *p* value was calculated using Tukey’s multiple comparison test. When *p* > 0.05, differences were considered non-significant (ns); when *p* < 0.05, differences were considered to be significant (* *p* < 0.05, ** *p* < 0.01, *** *p* < 0.001, **** *p* < 0.0001, respectively).

## Data Availability

Not applicable.

## Supplementary Materials

The following supporting information can be downloaded at: https://www.mdpi.com/article/10.3390/gels8120804/s1, The synthesis, HPLC profile, mass spectrum and ^1^H NMR spectrum of FmocFFRR, preparation of FmocFFRR hydrogel, cell attachment experiments, cell encapsulation experiments, and immunofluorescent staining of ALP and COL1 were listed in the supporting information.
