# Supplementary material for: Hydroxyapatite-Tethered Peptide Hydrogel Promotes Osteogenesis
Source: Gels. 2022 Dec 8;8(12):804. doi: 10.3390/gels8120804 (PMC9777555; doi:10.3390/gels8120804)
Supplement: Supplementary file 1 [file gels-08-00804-s001.zip › gels-2033972-supplementary.pdf]

Supporting Information

Hydroxyapatite-Tethered Peptide Hydrogel Promotes Osteogenesis

Hongwen Yu [1,2,3], Jiaqi Song [3], Xianpeng Zhang [3], Kuo Jiang [2], Hong Fan [2], Yibing Li [2], Yuanting Zhao [2], Shichang Liu [1,2] *, Dingjun Hao [1,2] *, Guanying Li [3] *

[1] *The Second Clinical Medical School, Shaanxi University of Chinese Medicine, Xianyang 712046, China*

[2] *Honghui Hospital, Xi'an Jiaotong University, Xi'an 710054, China*

[3] *School of Basic Medical Sciences, Xi'an Jiaotong University, Xi'an 710061, China*

*** Correspondence:**

Corresponding Authors

Shichang Liu: feitianyu1985@163.com;

Dingjun Hao: hdj_hhyy@163.com;

Guanying Li: guanyingli@xjtu.edu.cn.

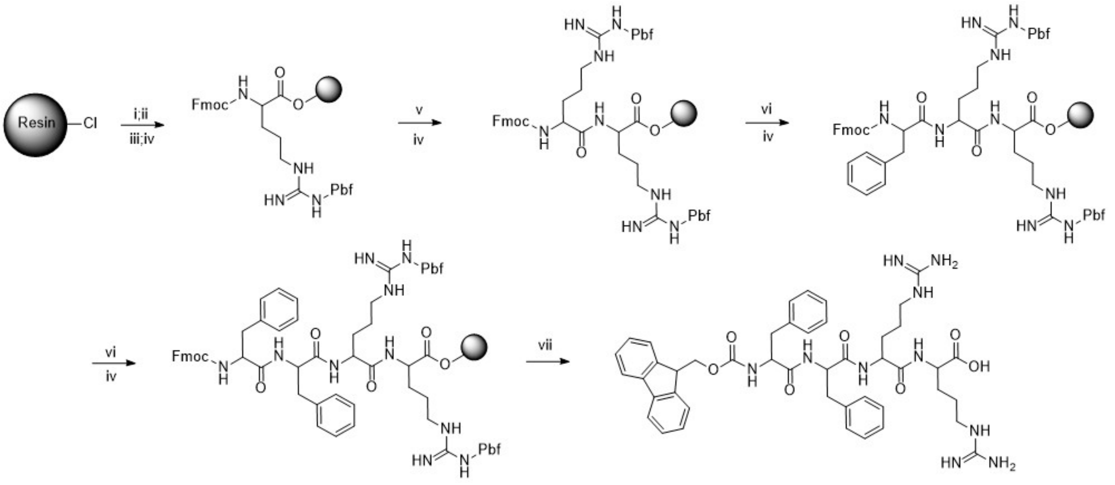

Scheme S1. Synthesis of peptide FmocFFRR. Reagents and conditions: (i) DCM; (ii) Fmoc-Arg (Pbf)-OH, DIEA, DMF; (iii) DIEA: MeOH: DCM (5:15:80); (iv) 5%Piperazine, 2%DBU, DMF; (v) Fmoc-Arg (Pbf)-OH, HBTU, DIEA, DMF; (vi) Fmoc-Phe-OH, HBTU, DIEA, DMF; (vii) 95%TFA + 2.5% Triisopropylsilane (TIS) + 2.5% H_2O.

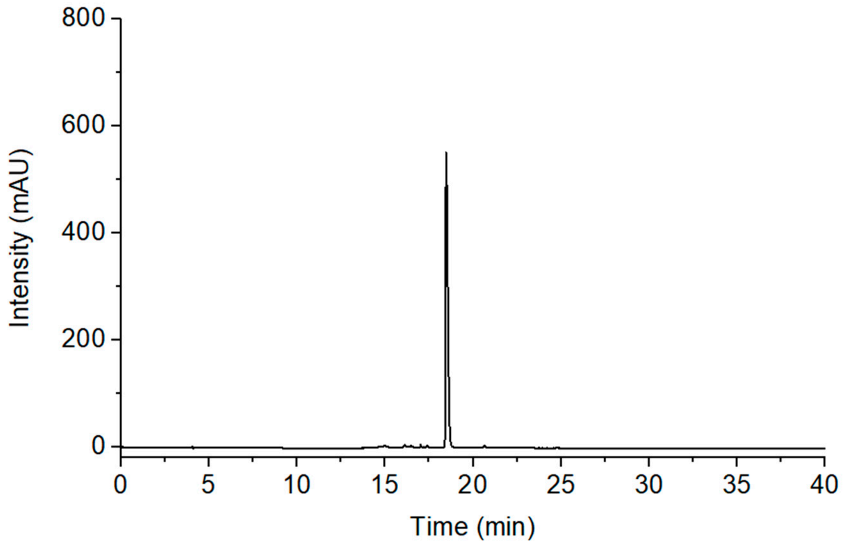

Figure S1. HPLC profile of FmocFFRR eluted with acetonitrile (ACN) and water containing 0.05% TFA. Detection wavelength: 280nm.

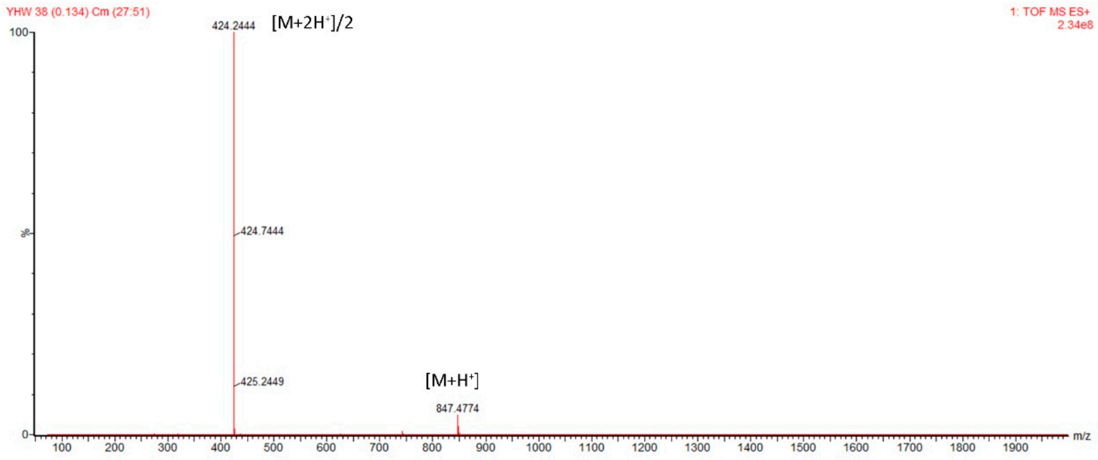

Figure S2. High-resolution mass spectrum of FmocFFRR in MeOH.

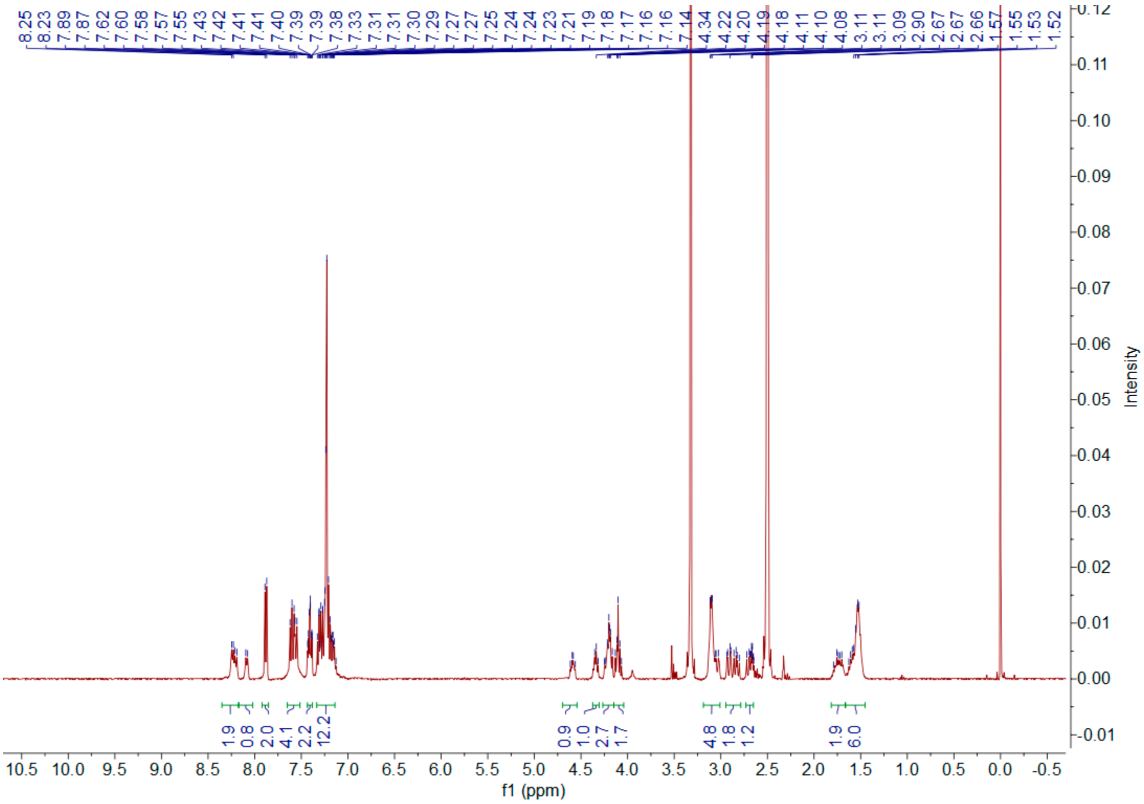

Figure S3. ^{1}H NMR spectrum of FmocFFRR in DMSO-d6, 400MHz.

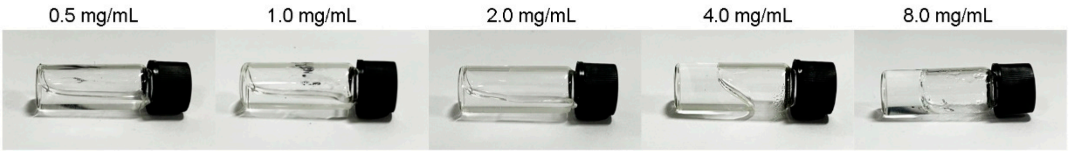

Figure S4. Optical images of FmocFFRR dissolved in ddH_2O at different concentrations.

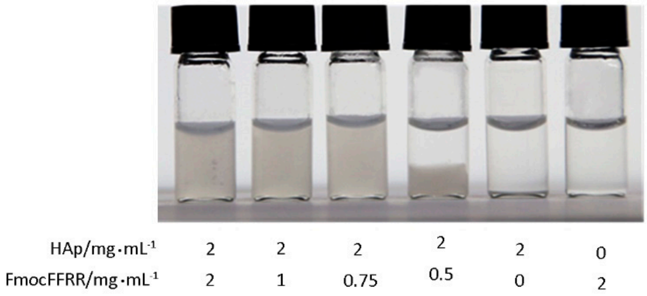

Figure S5. Optical images of 2mg/mL HAp particles in ddH_2O dispersed in FmocFFRR at different concentrations for 48h.

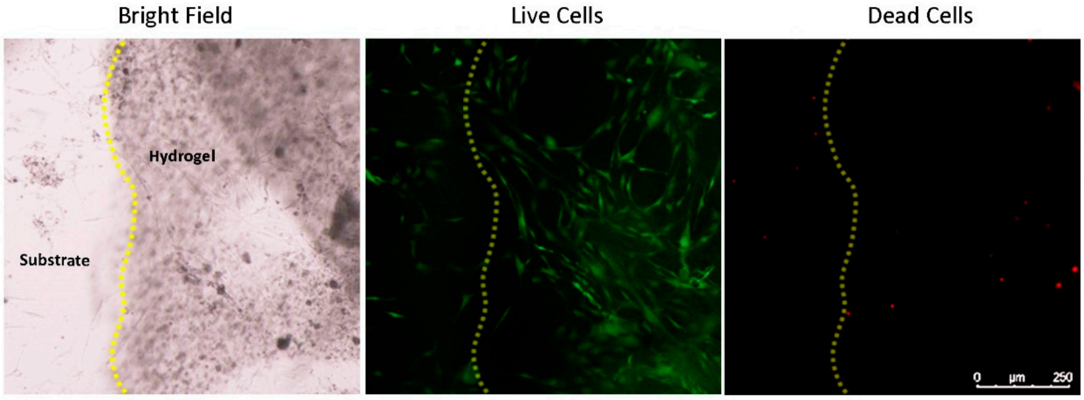

Figure S6. MC3T3-E1 cells were seeded on a 24-well plate that was pre-coated with FmocFFRR/ HAp hydrogel (2.5/5.0, mg/mL). After culturing overnight for attachment cells were stained with a Live-dead staining kit and captured on a fluorescent microscope. Live cells were stained with Calcein AM (green) and dead cells were stain with PI (red).

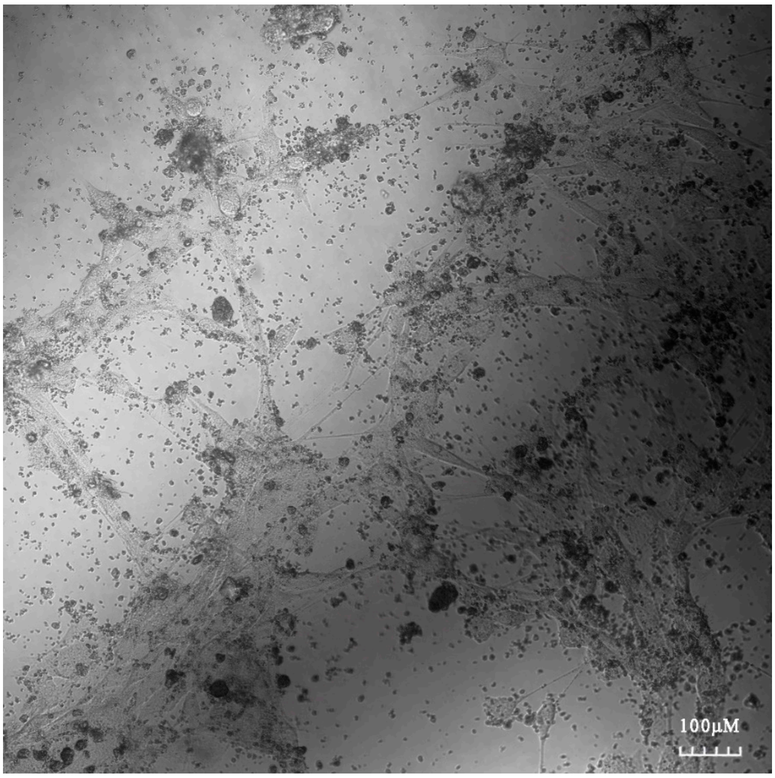

Figure S7. MC3T3-E1 cells were treated with 1mg/mL HAp particles for 24hours. After washed thoroughly with PBS, bright field image of cells was captured. Dotted particles indicate small HAp particles, and big clumps in dark color indicate HAp aggregates.

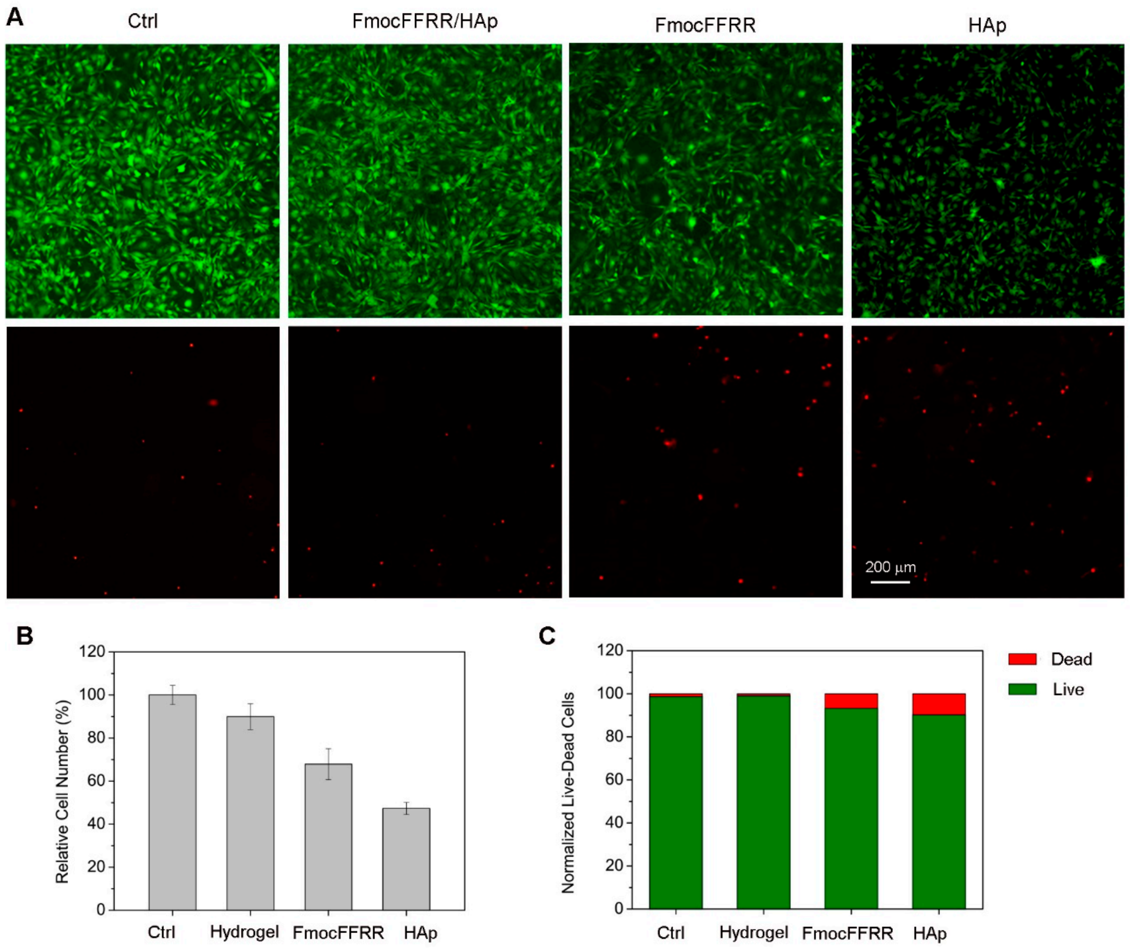

Figure S8. MC3T3-E1 cells were seeded on a 24-well plate and cultured overnight for attachment. Cells were treated with free HAp particles (5.0mg/mL), FmocFFRR assembly (2.5mg/mL) and FmocFFRR/HAp hydrogel (2.5/5.0, mg/mL) for another 48h. Cells were stained with a live-dead staining kit and captured on a fluorescent microscope. Live cells were stained with Calcein AM (green) and dead cells were stain with PI (red). (A) Fluorescent images of live cells (green) and dead cells (red). (B) Relative live cell numbers and (C) normalized live-dead cells were quantified.

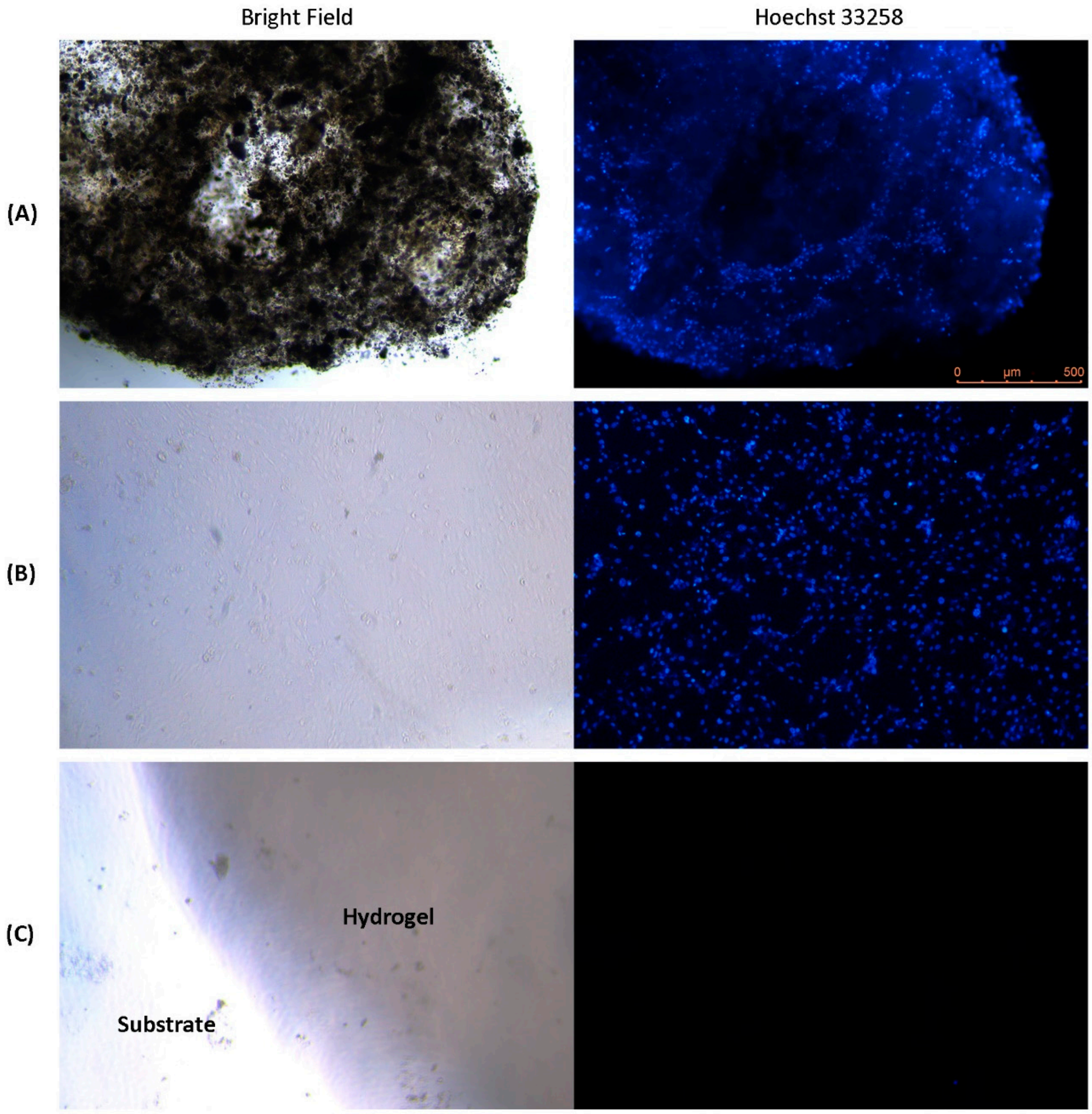

Figure S9. (A) FmocFFRR (final concentration 2.5 mg/mL) and HAp (final concentration 5.0 mg/mL) were mixed with MC3T3-E1 cells suspension for in situ gelation. After 48hour's of culturing in the basal culture medium, encapsulated MC3T3-E1 cells were stained with Hoechst 33258 and captured on a fluorescent microscope. For comparson, MC3T3-E1 cells attached to culture plate (B), and FmocFFRR/HAp (2.5/5.0, mg/mL) hydrogel without cells (C) were also stained with Hoechst 33258.

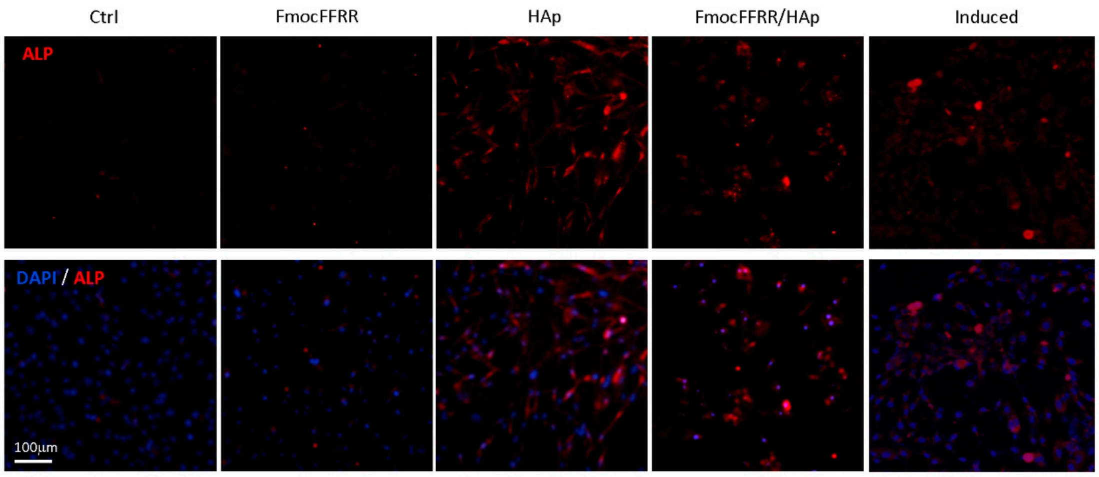

Figure S10. MC3T3-E1 cells were cultured with basal culture medium in FmocFFRR/HAp (5.0/10.0, mg/mL) hydrogel, free HAp particles (10.0 mg/mL), FmocFFRR assemblies (5.0 mg/mL), or treated with osteoinduction medium (Induced) for 7 days and immunostained with anti-ALP antibody to evaluate the expression of ALP. Cell nuclear were also stained with DAPI.

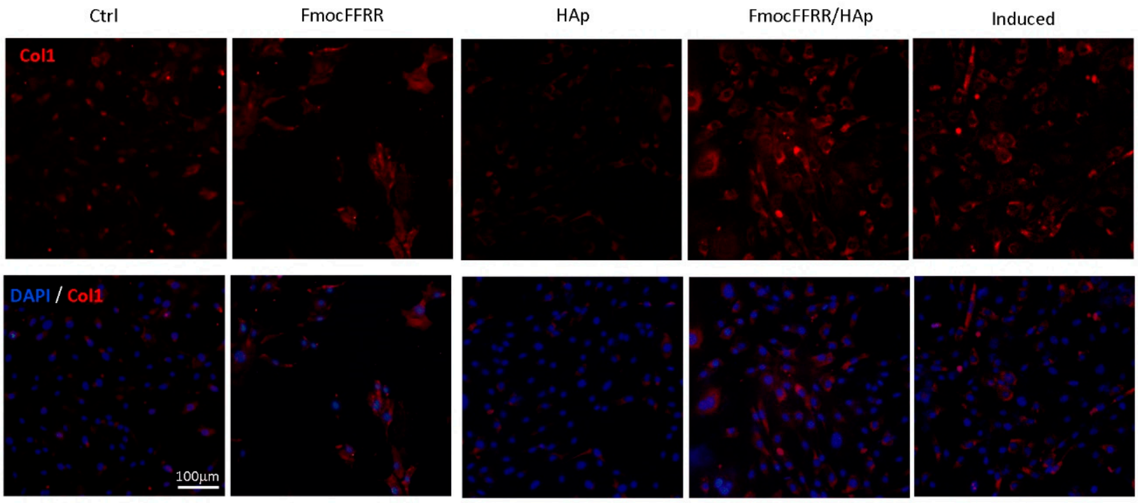

Figure S11. MC3T3-E1 cells were cultured with basal culture medium in FmocFFRR/HAp (5.0/10.0, mg/mL) hydrogel, free HAp particles (10.0 mg/mL), FmocFFRR assemblies (5.0 mg/mL), or treated with osteoinduction medium (Induced) for 7 days and immunostained with anti-COL1 antibody to evaluate the expression of type-1 collagen. Cell nuclear were also stained with DAPI.
